# Fas-Related Apoptosis of Peritoneal Fluid Macrophages in Endometriosis Patients: Understanding the Disease

**DOI:** 10.1155/2017/3175394

**Published:** 2017-11-01

**Authors:** Marek Gogacz, Krzysztof Gałczyński, Małgorzata Wojtaś, Izabela Winkler, Aneta Adamiak, Katarzyna Romanek-Piva, Tomasz Rechberger, Jan Kotarski

**Affiliations:** ^1^II Department of Gynecology, Medical University of Lublin, Ul. Jaczewskiego 8, 20-954 Lublin, Poland; ^2^Oncology Centre of Lublin, Ul. Jaczewskiego 7, 20-090 Lublin, Poland; ^3^I Department of Gynecology, Medical University of Lublin, Ul. Staszica 16, 20-081 Lublin, Poland

## Abstract

Recent studies of the peritoneal cavity environment in endometriosis demonstrate quantitative and qualitative changes in the cells responsible for cell-mediated immunity. Such changes may have led to disturbances in the surveillance, recognition, and destruction of misplaced endometrial cells and might have, in fact, brought about the disease. The aim of the study was to assess CD95 (Fas) expression on (activated) peritoneal fluid (PF) macrophages, as well as to ascertain soluble Fas (sFas) concentration in the PF of endometriosis patients, as compared to the nonendometriotic group. The concentration of leukocytes in the PF, the percentage of cells expressing CD45^+^/CD14^+^, and the percentage of PF macrophages expressing the HLA-DR antigen were significantly higher in patients with stages I and II endometriosis. The percentage of Fas- (CD95^+^-) expressing macrophages was significantly higher in all stages of the disease, in comparison with controls. Moreover, the concentration of sFas in the PF of patients with moderate and severe endometriosis was significantly higher, as compared to the reference group. The high number of immune cells in PF in early stage endometriosis and their increased susceptibility to apoptosis confirm the role of the impaired peritoneal environment and immune defects in the development and progression of the disease.

## 1. Introduction

Endometriosis is characterized by the presence of endometrial-like tissue outside the uterus [1, 2]. From the last decade of the 19th century, onwards, many attempts have been made to develop a definitive theory for explaining the pathogenesis of the disease, but its origin still remains controversial and poorly understood [3–6]. Still, current theory holds that retrograde menstruation, first described by Sampson, serves as a mechanism for the transportation of endometrial tissue which subsequently implants and proliferates at the ectopic sites. However, while the retrograde flow of blood into the peritoneal cavity is observed in 76–90% of all women, endometriosis develops only in 15% of all women of reproductive age. This suggests the involvement in the pathogenesis of endometriosis of other factors, such as impaired peritoneal environment [7].

It is well recognized that cell-mediated immune response contributes to the elimination of foreign antigens and misplaced autologous cells such as ectopic endometrial cells [8]. This mechanism may, hence, also protect most women against endometriosis. Therefore, alterations in cell-mediated immunity may promote the development of the disease [9]. Furthermore, in the peritoneal cavity, the peripheral blood monocytes (PBM), as well as the peritoneal macrophages (PM), represent the primary line of host defense against endometriosis. These multifunctional immune cells may also control the activity of other cells and play a key role in the pathogenesis of endometriosis [10, 11].

Apoptosis is one of the mechanisms responsible for the hemostasis of the immune system which eliminates activated and inactivated cells. Several pro- and antiapoptotic factors have been identified [12, 13]. The Fas antigen (APO-1/CD95) is a glycosylated 48 kDa surface protein and is a member of the tumour necrosis factor/nerve growth factor receptor superfamily with proapoptotic properties [14]. Fas is expressed in various human tissues, including immune cells, and in many different tumour cells [15–18]. The triggering of Fas by its ligand (FasL) or by certain anti-Fas agonistic antibodies results in rapid induction of programmed cell death in susceptible Fas-bearing cells.

The expression of Fas cell surface protein is enhanced by the activation of lymphocytes by IFN-*γ* and TNF [19]. An enzymatically exfoliated extracellular domain of Fas receptor (sFas) is present in the body fluids, but its role is not well known yet. Probably, sFas competes with membrane Fas for the binding of the Fas ligand. The malfunction of the Fas system induces immune disorders and promotes the occurrence of autoimmune disease, whereas its exacerbation may cause tissue destruction.

Recent studies of the peritoneal cavity environment in women with endometriosis demonstrate quantitative and qualitative (functional) changes in monocytes/macrophages and natural killer cells, as well as the cytotoxic T and B lymphocytes responsible for cell-mediated immunity. The observed changes may disturb the surveillance, recognition, and destruction of misplaced endometrial cells and, hence, induce impairment of immune cell-mediated response in the peritoneal cavity. This situation possibly leads to endometriosis.

The aim of the study was to assess CD95 (Fas) expression on (activated) peritoneal fluid (PF) macrophages, as well as to ascertain soluble Fas (sFas) concentration in the peritoneal fluid of patients with endometriosis, as compared to the nonendometriotic group.

## 2. Material and Methods

### 2.1. Ethics and Informed Consent

The study was conducted at II Department of Gynecology, Medical University of Lublin (Poland). The protocol was reviewed and approved by the Institutional Review Board of the Medical University of Lublin before the start of the study. All potential participants were provided with a verbal and written informed consent regarding the reasons for the study and listing potential adverse effects. Each woman, by signing the informed consent, acknowledged and accepted the provided information.

### 2.2. Patients

The study group consisted of 26 women of reproductive age with endometriosis as confirmed by histological results. The stage of the disease was assessed according to the revised American Society of Reproductive Medicine classification [20]. Stage I, stage II, and, together, stages III and IV were found in 11, 10, and 5 patients, respectively. The control group consists of 18 women of reproductive age who had undergone surgery due to benign noninflammatory and nonendometrial ovarian cysts. All patients had regular ovulatory cycles varying in duration between 26 and 33 days. Oral contraceptives, hormone-releasing intrauterine devices, or ovulation-inducing drugs were not used for at least 3 months before surgery.

### 2.3. Peritoneal Fluid Collection

Peritoneal fluid (PF) was collected from 44 patients during a laparoscopy that was performed due to infertility or the presence of a benign noninflammatory mass of ovary/ovaries in the midfollicular phase of the cycle (6th–14th day of the cycle). The obtained fluid (3–5 ml) was aspirated under direct vision, from either the Douglas or uterovesical pouch, with a nylon catheter, at the beginning of the surgery, after trocar introduction, and was placed in sterile heparinized tubes (Greiner). Any contamination of the fluid with blood from injured vessels after trocar insertion excluded patients from the analysis.

### 2.4. Concentration of Immune Cells in Peritoneal Fluid

After homogenous dispersion of immune cells, its concentration was counted in Thom's hemocytometer and recorded individually (per patient).

### 2.5. Preparation of the Cells

Mononuclear cells were isolated on density gradient medium (Polymorphrep, Nycomed, Norway) by centrifugation at 600*g*, for 25 minutes, at room temperature. After centrifugation, PF supernatant was aspirated and stored until analysis. The cell pellet from the interphase was then removed, washed twice in phosphate-buffered saline (PBS, Biomed, Lublin) containing 1% bovine serum albumin, and resuspended in polyethylene tubes at 0.5 × 10^6^ cells each. The prepared cells were incubated with monoclonal antibodies for 30 minutes at 4°C and washed twice afterwards.

### 2.6. Phenotyping of Peritoneal Fluid Immune Cells

Double-colour immunofluorescence studies were performed using combinations of (FITC/PE) monoclonal antibodies—conjugated with fluorescein isothiocyanate (FITC) and phycoerythrin (PE). The following antibody combinations were used:

- IgG1 FITC/IgG2a PE (Ortho Diagnostic Systems) (Germany) as negative control
- CD45/14 (Ortho Diagnostic Systems) (Germany)
- CD14/HLA-DR (Ortho Diagnostic Systems) (Germany)
- CD3/19 (Ortho Diagnostic Systems) (Germany)
- CD 3/16/56 (Ortho Diagnostic Systems) (Germany)
- CD95 PE (Immunotech)

### 2.7. Flow Cytometric Analysis of Expression of CD Antigens

All samples were measured on a Cytoron Absolute flow cytometer (Ortho Diagnostic System). In this part of the experiment, 10000 cells were collected and analyzed per test for fluorescence. The samples were first stained with propidium iodide, and dead cells that showed staining were excluded from further analysis. In this experiment, the mononuclear cell subpopulation was represented as a percentage of the entire CD45-positive cell population, while PF macrophages were determined by CD14 monoclonal antibody content. To recognize subpopulations of T/B lymphocytes and natural killer cells (NK cells), CD3/19 and CD3/16/56 monoclonal antibodies were used. Active cells were detected by applying HLA-DR monoclonal antibodies. Antigen density within the cell was then estimated via mean fluorescence intensity. In order to quantitate the levels of fluorescence, the mean fluorescence intensity (MFI) of antigen-positive cells was calculated. To determine the fluorescence intensity of the stained cells, the logarithmic fluorescence channel intensity was converted to arbitrary units based on the Immuno Count 2.0 software. The MFI of the CD-positive histogram was measured from the upper limit of the negative control. Antigen-positive cells were compared to the appropriate FITC or PE-conjugated mouse IgG1/IgG2 control cells.

### 2.8. Concentration of Soluble Fas in PF

PF supernatants were collected immediately after centrifugation and stored at −700°C until analysis, not longer than 8 months. In the analysis, sFas concentration in the stored samples was measured with a sandwich enzyme-linked immunoassay (ELISA) kit (Chemicon, USA), according to the manufacturer's instruction. The data are shown as the mean values of duplicate samples. The sensitivity of the test was 0.1 U/l, and the range of standards was 0–15 U/l.

### 2.9. Statistical Analysis

The Statistica 5 test program was used to compare differences between the study and the reference groups. Data are expressed as means ± SD, and *p* < 0.05 was considered as significant.

## 3. Results

### 3.1. Concentration of Immune Cells in PF and Percentage of Peritoneal Fluid Macrophages (CD45^+^/CD14^+^)

#### 3.1.1. Immune Cell Concentration

In women with endometriosis, significantly higher concentrations of PF leukocytes (*p* < 0.001) were found, in comparison to the reference group (Table 1). Moreover, the concentration of leukocytes in the PF of patients with stage I (2490 cells/mm^3^ ± 1116) and stage II (2210 cells/mm^3^ ± 1099) of endometriosis was significantly higher (*p* < 0.001 and *p* = 0.003, resp.) when compared to the reference group (1231 cells/mm^3^ ± 420). No statistical difference (*p* > 0.05) was found in patients with endometriosis stage III/IV (1620 cells/mm^3^ ± 377), in comparison to the reference group, but it is hard to draw conclusions from this result due to small sample of cases with advanced stages of endometriosis recruited to the study.

#### 3.1.2. Macrophages

In patients with endometriosis, a significantly higher (*p* < 0.0001) percentage of PF cells expressing CD45^+^/CD14^+^ was found, as compared to the reference group. In addition, a significantly higher percentage of PF macrophages was detected in women with stages I and II (*p* < 0.001 and *p* < 0.001), but not in patients with stage III/IV (*p* > 0.05) endometriosis, when compared to the reference group (Table 1). However, the mean fluorescence intensity of CD14^+^ antigen on PF macrophages did not differ in patients with endometriosis, when compared to the reference group (Table 1) and between patients with different stages of the disease.

Beyond the aforementioned, T-lymphocytes, CD3^+^/CD19, and NK cells, CD3^−^/CD16/56^+^, in the PF of patients with endometriosis were found to be as much as 16.3% of all examined immune cells. This is in contrast to the same within 41.8% of all examined cells in the reference group. Moreover, B lymphocytes, CD3^+^/CD19^+^, were found to amount to less than 0.5% of the entire examined PF leukocyte population in both the study and the reference groups.

### 3.2. Expression of HLA-DR (a Marker of Activation) on PF Mononuclear Cells

In the endometriotic patients, a significantly higher (*p* < 0.01) percentage of PF macrophages expressing the HLA-DR antigen (HLA-DR^+^ macrophages) was observed, as compared to the reference group: 85.4% ± 7.3 versus 62.2% ± 31.1, respectively. However, the expression of HLA-DR was significantly higher only in patients with stages I (*p* = 0.02) and II (*p* = 0.04) of endometriosis and did not differ in patients with stage III/IV of endometriosis (*p* = 0.07), when compared to the reference group (Table 2). Furthermore, the MFI of the HLA-DR antigen in macrophages obtained from endometriotic women was statistically higher (*p* = 0.01), as compared to the reference group: 148.0 ± 20.4 versus 129.3 ± 28.0, respectively. No significant differences were found when patients with different stages of the disease (I, II, and III/IV) were compared to the reference group (Table 2).

### 3.3. Expression of CD95 (Fas) Antigen on PF Macrophages

**Figure 1:**
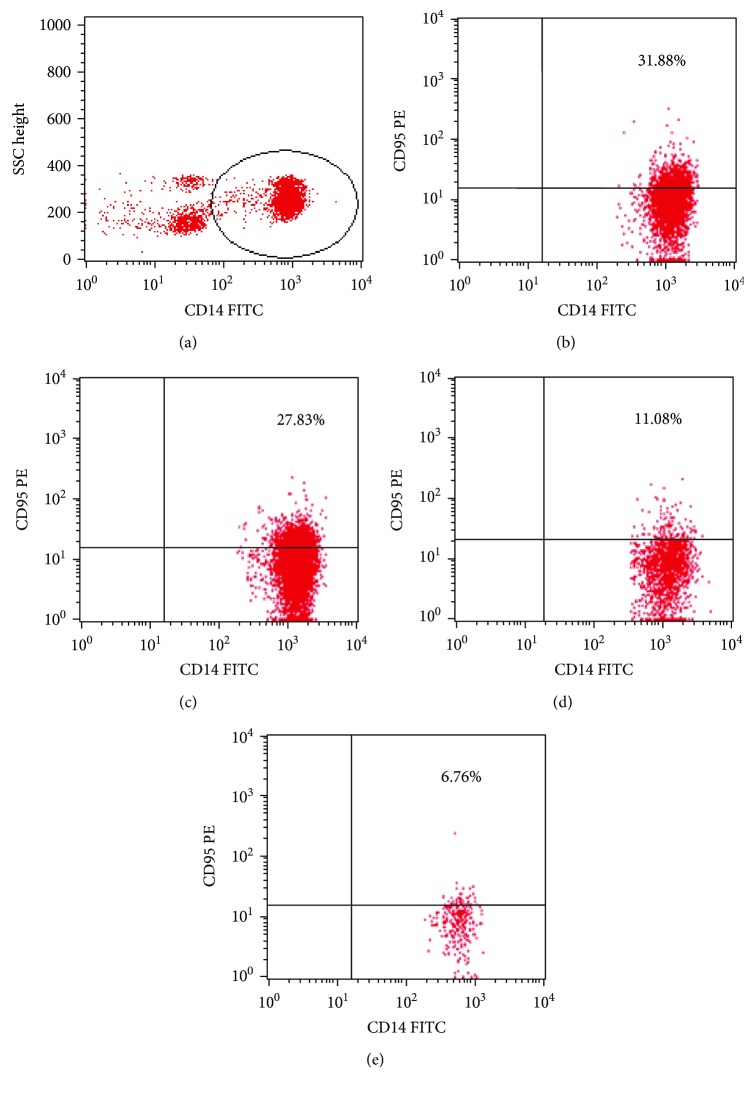
Representative dot plots, illustrating our analysis method for the identification of CD14^+^ cells with CD95 (Fas) expression. (a) An acquisition gate was drawn around the CD14^+^ cells. (b–e) Next, the gated events were analyzed for CD95 PE. The dot plots (b–d) show representative types of CD95 expression in CD14^+^ cells from patients with endometriosis. The dot plot (d) shows the representative type of CD14^+^CD95^+^ cells from the reference group.

Fas (CD95^+^) was expressed on 20.1% ± 15.7 of all observed macrophages, in patients with endometriosis, whereas only 6.6% ± 10.1 of all macrophages from the reference group expressed CD95 superficially (Figure 1). The observed differences in Fas expression were statistically confirmed in all stages of endometriosis. With regard to MFI, no statistical differences between study groups regardless of the stage of the disease (I, II, and III/IV) and the reference group were observed (Table 2).

### 3.4. Soluble Fas Concentration in PF

The concentration of the soluble form of Fas (sFas) in the PF of patients with II and III/IV stage endometrioses was 9.9 ± 7.4 and 16.2 ± 13.1, respectively, which was significantly higher than that found in the reference group: 5.5 U/l ± 4.4 (Table 2).

## 4. Discussion

Numerous studies showed systemic and/or local (qualitative and quantitative) changes in patients with endometriosis. This is the first study concurrently evaluating the concentration/expression of opposite markers related to activation and apoptosis on PF macrophages (proliferative and proapoptotic) in patients with endometriosis and healthy controls. The immune background of endometriosis is well established, and much data regarding PF leukocyte components are available, yet some are contradictory. In the presented study, for example, significantly higher concentrations of macrophages in the PF of patients with endometriosis were found. This comprised more than 80% of all PF leukocytes. This observation is in agreement with data published by Weinberg et al., Haney et al., Halme et al., and Hill [21–24]. Contrary to our data, Awadalla et al. and Zeller et al. observed lower percentages of PF macrophages in women with endometriosis [25–27].

Interestingly, in several studies, in women with endometriosis, significantly higher concentrations of PF macrophages were found, even when compared to cases with abdominal infections. In the latter condition, a predominance of neutrophils has been observed (85% of PF leukocytes). The mechanism responsible for such cell distribution is still unknown.

Akoum et al. demonstrated that in women with endometriosis, peritoneal macrophages had an increased capacity to secrete MCP-1 (monocyte chemotactic protein-1) [27]. This mechanism might exacerbate peritoneal inflammation and promote the growth of endometrial implants. Tao et al. found that peritoneal MCP-1 plays an important role in the pathogenesis of infertility in the early stage of endometriosis [28]. In our study, the highest percentage of macrophages, as well as the highest level of leukocytes, was in the group of women with I and II stages of the disease. Such a situation could be the result of the mobilization of the local immune response in minimally advanced endometriosis. The question is still unanswered why the specific immunological response is developed only in the early stage of endometriosis and is weakened in more advanced stages. We put forward that there is a critical point in the natural history of endometriosis when self-defense mechanisms are switched on to avoid the autodestruction of the body.

Worth mentioning is the observation that in the group of women with endometriosis, macrophages did not only prevail amongst the immune cells in the peritoneal fluid but that more than 84% of these also activated and presented the expression of HLA-DR—a recognized activation marker of macrophages. Our data show that the expression of HLA-DR on PF mononuclear cells in women with endometriosis is significantly higher in stages I and II of the disease. We thus surmise that cell-mediated immunity is the first line of immune response in endometriosis, but still it is not obvious if this reaction is sufficient to obtain peritoneal homeostasis. It, hence, may only indirectly take part in the antigen presentation to T cells.

Activated macrophages are capable of secreting several biological substances. This process could be responsible for inducing the growth of endometriotic lesions in women with endometriosis. This theory tends to support the works of Halme et al., Koutsilieris et al., Olive et al., and Hammond et al. [29–32]. They held that PF macrophages secrete biological substances which promote the proliferation of endometrial cells. Moreover, concentrations of this substances increase with the advance of the disease [33, 34]. The expression of HLA-DR on macrophages is crucial for the induction of a humoral immune response. In accordance with our observation that in the group of women with endometriosis, the percentage of activated macrophages was twofold higher than that in the control group, we opine that the function of macrophages is also increased. Still, many authors have found a dysfunction of PF macrophages in endometriosis, but the etiology is of yet unknown [9, 35, 36]. Dmowski et al. suggest that this results from a congenital or acquired defect of the immune system. Of note, in recent years, many studies have seen the inhibiting influence of environmental or immunosuppressant substances in the PF of women with endometriosis [8].

Many authors have also emphasized the dual role of macrophages in the pathogenesis of the disease. Firstly, they intensify the immune response leading to the elimination of pathologic lesions. Secondly, and in contrast, they promote the growth of endometriotic implants. These two processes form a vicious circle and could be responsible for the developing of endometriosis. Further studies of this issue, however, are still required.

Many studies have also evaluated the role of immune activation in women with endometriosis (i.e., bcl- and bax-gene activation in PF macrophages); however, only a few studies have dealt with the issue of macrophage apoptosis in endometriosis and have assessed Fas antigen expression on PF macrophages in the disease [37, 38]. Our study generated an estimate of the presence of the Fas antigen on the macrophage surface.

Some authors have suggested that proapoptotic receptors and their ligands on the surface of immune and endometrial cells play crucial roles in the pathogenesis of endometriosis [39]. In the presented study, in the group of women with endometriosis, the level of the Fas receptor was statistically higher than that in the control group. Moreover, the expression of the Fas receptor increased with the stage of the disease. Indeed, the percentage of PF macrophages with the Fas receptor in patients at stage III/IV of endometriosis was more than twofold higher than that in stage I. This situation could be the result of a higher concentration of TNF*α* in the peritoneal fluid of women with endometriosis [40–44].

The presence of TNF*α* upregulates the Fas receptor in the cells, and it brings about Fas-induced apoptosis. We suspect that activated macrophages are the source of TNF*α* which begins a cascade of events finally leading to the increased synthesis of IFN-*γ*. This, on the one hand, enhances the cytotoxicity of NK cells and macrophages and, on the other hand, promotes cell self-destruction.

Garcia-Velasco et al. found a stage-dependent decreased expression of the Fas ligand (FasL) on the ectopic endometrium in women with endometriosis [7]. The expression, they put forward, was induced by dose-dependent macrophage-derived factors such as TGF-*β* and PDGF. Furthermore, they held that the interaction between endometrial cells and the extracellular matrix (which contains laminin, fibronectin, and collagen IV) upregulates FasL expression. This may protect the endometriotic cells from attack by activated T lymphocytes, and the Fas-FasL mechanism may also allow eutopic endometrial cells to escape from immune surveillance. The lower activity and concentration of the immune cells in the peritoneal fluid of women with endometriosis appear to confirm this scenario [25, 26, 45].

It is worth mentioning about the effect of Danazol and the aromatase inhibitors on the immune system in endometriosis. Such drugs normalize the peritoneal concentration of TNF*α* and increase the number and cytotoxicity of immune cells acting against the ectopic endometrium [1]. In our work, at the advanced stages of endometriosis, a significantly higher percentage of PF macrophages with Fas expression was observed (accompanied by the lowered concentration of soluble forms of the receptor), but the reliability of this finding is limited by the small number of analyzed cases with advanced endometriosis.

Our data show that the level of soluble forms of Fas in the group of women with stages II and III/IV of endometriosis is statistically higher than that in the reference group. The same observation was made by Linghu et al. [46]. In a related work, the concentration of sFasL in the serum and peritoneal fluid of women with moderate to severe endometriosis was elevated when compared to that of women with minimal and mild endometriosis [47]. In this study, the sources of soluble FasL in the peritoneal fluid were thought to be the ectopic endometrial cells and the peritoneal leukocytes. Still, other studies report that matrilysin (uterine metalloproteinase) generates the active soluble form of FasL [48, 49].

The presence of both forms of the Fas ligand (soluble and membrane-bound) in the peritoneal fluid of women with endometriosis seen in cited works, in comparison with our results, may suggest increased immune cell apoptosis in the peritoneal cavity. The deficiency of immune cells, therefore, must lead to local immune dysfunction, to incomplete immune response, and, consequently, to the development of the disease. The significantly higher percentage of macrophages expressing the Fas receptor supports the hypothesis that the PF components of women in endometriosis may promote the development of the disease.

## 5. Conclusion

An imbalance between apoptosis and activation/surveillance/proliferation of immune and endometrial cells may play an important role in the pathogenesis of endometriosis.

The high number of immune cells in the peritoneal fluid and their increased susceptibility to apoptosis in early-stage endometriosis emphasize the role played by both an impaired peritoneal environment and immune defects in the development of the disease.

## Figures and Tables

**Table 1:** The mean (±SD) concentration of leukocytes, percentage of macrophages, and MFI of CD14 antigen in peritoneal fluid.

	Endometriotic patients (*n* = 26)	Stage I (*n* = 11)	Stage II (*n* = 10)	Stage III/IV (*n* = 5)	Reference group (*n* = 16)
Concentration of PF leukocytes (cells/mm^3^)	2215 ± 1030^∗^	2490 ± 1116^∗^	2210 ± 1099^∗^	1620 ± 377 NS	1231 ± 420
Percentage of PF macrophages CD45^+^/CD14^+^ (%)	83.2 ± 7.8^∗^	84.2 ± 6.5^∗^	85.4 ± 5.58^∗^	67.6 ± 11.6 NS	57.7 ± 12.4
MFI of CD14 antigen	132.8 ± 32.4 NS	119.4 ± 27.5 NS	144.5 ± 28.2 NS	138.9 ± 45.1 NS	135.9 ± 19.9

^∗^Statistically significant compared to reference group (*p* ≤ 0.05). NS: statistically nonsignificant.

**Table 2:** Soluble form of Fas (sFas) antigen concentration in peritoneal fluid and percentage of macrophages expressing CD95 (CD95^+^) and HLA-DR (HLA-DR^+^) antigens.

	Endometriotic patients (*n* = 26)	Stage I (*n* = 11)	Stage II (*n* = 10)	Stage III/IV (*n* = 5)	Reference group (*n* = 16)
CD95^+^ macrophages (%)	20.1 ± 15.7^∗^	17.6 ± 15.2^∗^	16.6 ± 7.8^∗^	37.4 ± 19.8^∗^	6.6 ± 10.1
MFI of CD95 antigen on macrophages	101 ± 17.5 NS	101.5 ± 13.5 NS	108.9 ± 17.8 NS	90.7 ± 25.6 NS	98.9 ± 24.2
HLA-DR^+^ macrophages (%)	85.4 ± 7.3^∗^	85.6 ± 7.1^∗^	84.0 ± 7.7^∗^	89.0 ± 7.1 NS	62.2 ± 31.1
MFI of HLA-DR antigen on macrophages	148.0 ± 20.4^∗^	146.9 ± 14.1 NS	145.8 ± 14.6 NS	156.6 ± 39.3 NS	129.3 ± 28.0
sFas concentration in PF (U/l)	9.0 ± 7.4 NS	4.7 ± 3.1 NS	9.9 ± 7.4^∗^	16.2 ± 13.1^∗^	5.5 ± 4.4

^∗^Statistically significant in relation to the reference group. NS: statistically nonsignificant.
